# Finite Element Approach for Rheological Behavior in Colloidal Electrolytes in Lithium-Ion Battery Performance

**DOI:** 10.1021/acsomega.4c04445

**Published:** 2024-08-07

**Authors:** Ahsan Raza, Tareq Manzoor, Shaukat Iqbal, Tauseef Anwar, Adeel Ashraf, Habib Ullah Manzoor

**Affiliations:** † School of Systems and Technology, University of Management and Technology, Lahore 54000, Pakistan; § Department of Physics, University of Education, Joharabad Campus, Lahore 54000, Pakistan; ‡ Energy Research Centre, COMSATS university Islamabad, Lahore 54000, Pakistan; ∥ James Watt School of Engineering, University of Glasgow, Glasgow G12 8QQ, U.K.

## Abstract

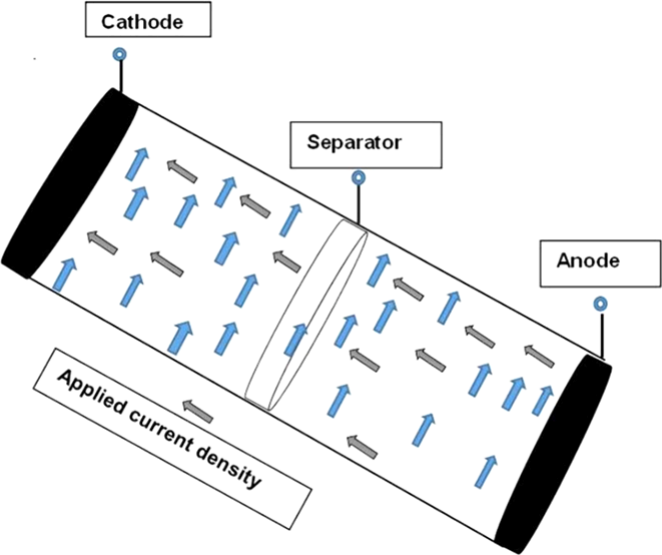

The main implication of articulating electrolyte performance is studying the energy density, charging aspects, formation of precipitates, thermal fluctuations during charging–discharging, and safety of batteries against fire or spark. One of the most significant aspects is the ability to design colloidal electrolytes that can enhance the overall performance of batteries along with dealing with all internal problems within a battery system. Through this optimization progression, the general performance and efficiency of Li-ion batteries can be improved. This work is presented in the study of the boundary value problem for rheological properties of colloidal electrolytes as a fourth grade fluid for lithium ion (Li-ion) batteries down a vertical cylinder. They have exceptional characteristics, such as low volatility and high thermal stability. The practical usage of the exact flow is restricted, as it involves very complicated integrals. The nonlinear problem that arises is solved by Galerkin’s finite element approach based on the weighted-residual formulation, which is used to find the approximate solutions of the fourth-grade problem. This approach utilizes a piecewise linear approximation using linear Lagrange polynomials. Convergence of the solutions, which briefly describes the flow characteristics, includes the effects of the emerging parameters. The results obtained after implementation are not restrictive to small values of the flow parameters. Numerical studies have shown the superior accuracy and lesser computational cost of this scheme in comparison to collocation, the homotopy analysis method, and the homotopy perturbation method. The impact of the relevant parameters is examined through graphical results after implementation of a number of iterations.

## 1 Introduction

Lithium-ion (Li-ion) based energy storing devices or batteries have experienced swift development in terms of applications in portable microelectronic maneuvers, electric vehicles (EVs), and micro grid-related power storage. Due to the necessity of an electrolyte, some chemicals, such as gel polymer electrolytes (GPEs), have garnered significant research attention due to their unique characteristics as liquid, semisolid, and solid electrolytes. These elements exhibit high ionic conductivity, surpassing 10^–4^ S cm^–1^, along with a wide electrochemical window, favorable chemical properties, thermal stability, and compatibility with electrodes during cycling. The specific requirements and essential properties of GPEs for lithium-ion battery (LIB) applications have been extensively discussed. Additionally, the transport mechanism of Li^+^ ions has been thoroughly explained. Recognizing the immense potential of GPEs in LIBs, recent advancements in GPEs based on various polymer types have been meticulously reviewed.^1−7^ As of right now, the most common kind of Energy Storage and Delivery Systems (EESDs) in daily life are rechargeable LIBs. Electric cars and consumer devices are powered by them on a large scale. Ionic liquids that contain lithium ions (Li+) move through the electrolyte from the cathode to the anode during the charging process of lithium-ion batteries (LIBs), with the electrodes remaining separated thanks to the porous separator. Electrons also travel to the anode simultaneously but do so via a longer route in the external circuit. By utilizing flexible solid polymer electrolytes (SPEs), the safety concerns associated with Li-based batteries can be addressed and electronic devices can be made more manageable.^8−13^

This is accomplished by substituting the combustible nonaqueous liquid electrolytes with SPEs. In this regard, some high-efficiency electrolytic chemicals have gained importance. Currently, fire-protection related problems initiating from organic solvents have led researchers to choose complex fluids, whereas various chemicals being used as LIB electrolytes have been discussed in Table 1. The interface between the colloidal electrolyte in Li-ion batteries and solid structures, including electrodes and separators, can be investigated through finite element analysis. This investigation may allow for the modeling of electrolyte–electrodes interactions. The problems which exist in real-world scenarios based on the utilization of geometry, analytics and variable based mathematics include different factors, as signified throughout the social sciences, medicine, natural sciences, energy sector, medicine, business and bioengineering.^5−10^

**Table 1:** Various Chemicals Used as LIB Electrolytes

Sr. No.	Electrolyte	Type of Electrolyte	Investigated by/Ref
1	Lithium hexafluorophosphate (LiPF_6_) salt dissolved in organic carbonates	nonaqueous, rate performance,	(1)
2	Fluorinated electrolytes (e.g., 1.2 M LiPF_6_ in F-AEC/F-EMC/F-EPE (2/6/2))	nonaqueous	(2)
3	Li_2_B_12_F_12–*x*_H_*x*_ electrolyte with the additive of lithium difluoro(oxalato)borate (LiDFOB)	nonaqueous	(3)
4	LiNO_3_ aqueous electrolyte	Aqueous, rate performance	(4)
			(5)
			(7)
5	Li_2_SO_4_ aqueous electrolyte	Aqueous, rate performance	(8)
6	LiTFSI aqueous electrolyte	aqueous	(9)
7	([ETMIm][TFSI]) with imidazolium based ionic liquids as solvents	ionic liquid (IL) ionic conductivity	(10)
8	Poly(ethylene oxide) (PEO)	solid polymer cycle performance	(11)
9	Poly(methacrylate) (PMA)	solid polymer	(12)
10	Single-ion BAB triblock copolymer electrolytes	solid polymer	(13)
12	Poly(acrylonitrile) (PAN)/poly(methyl methacrylate (PMMA)/polystyrene (PS)	gel polymer	(15)
13	Poly(methacrylate) (PMA)/poly(ethylene glycol) PEG-based GPE	gel polymer	(16)

The combination of cathode and anode materials determines the high current density of a lithium-ion battery, but both electrodes can exhibit thermodynamic instability when exposed to most electrolytes. Aqueous and protic electrolytes are unsuitable due to their immediate reaction with lithium, leading to inflammation and hydrogen formation. Additionally, many common organic solvents decompose on the anode side during battery charging. To better understand the rheological properties of different battery components, the Finite Element Method allows for spatially resolved simulations and detailed analysis. This approach is especially important in colloidal electrolyte research, where material properties can vary between electrolytes due to factors such as concentration and temperature.^14−19^ The Finite Element Method (FEM) is a numerical method of solving systems of partial differential equations (PDEs). It reduces a PDE system to a system of algebraic equations that can be solved by using traditional linear algebra techniques. In a simple word, the FEM is a method for dividing up a very complicated problem into small elements that can be solved in relation to each other. The FEM has a very deep impact in the field of engineering, computational design, and mathematical physics and is utilized to resolve the complexity of these differential equations. The FEM is generally referred to for structural analysis, heat transfer, fluid dynamics, electromagnetic potential, and automobile engineering problems; this requires the solution of boundary value problems that exist in the partial differential equation. This method formulates the problem’s result in the form of algebraic equations, where the unknown function was approximated within the domain. In the FEM the problem is that we are considering subdividing a big system into smaller and simpler finite parts. These finite parts are then brought together again into a big system that models the entire problem. The FEM once utilized the variational technique to approximate the solution by minimizing the value of the error function. Numerical modeling and simulation have a very deep knowledge in the field of science, which uses different methodologies to achieve the optimal solution of the existing problems. Higher grade fluids for lithium ion batteries have been modeled in the homotopy perturbation method (HPM) and the traditional perturbation method to obtain the approximated result of the nonlinear equation of the fourth grade thin fluid of the outer surface of the cylinder.^20−25^ Scientists also discussed the flow problem of the third-grade thin film on a vertically moving belt by both the HPM and the traditional perturbation techniques and found that the HPM will overcome the many limitations of the traditional perturbation and also found the velocity, volume flux and average.^26−29^ They introduced the new nonlinear similarity transformation where they used the HPM for boundary-layer flow of a viscous fluid nonlinear axisymmetric stretching sheet where the HPM is used in the partial differential equation and the ordinary differential of nonlinear equations.^30−33^ Similarly, the analytic solution of the thin film flow of the fourth grade fluid down a vertical cylinder is obtained using the homotopy analysis method (HAM), and then compare the HPM results are compared with the HAM results; it is found that the HPM results are divergent for strong nonlinearity as compared to the HAM, which is a simple and efficient technique to control and handle the convergence result.^34−39^ So the same methodology is applied but on the slip effect of the thin film fluid of the fourth grade vertical cylinder where the nonlinear equation is solved by both an exact and the HAM techniques and their results are also compared.^40−45^ Marinca et al. proposed the optimal homotopy asymptotic method (OHAM) on the steady flow of the fourth grade fluid, but on the past porous plate, which does not depend on a very small and large parameter, which provides an easy way to control the convergence of the estimated series, and the series solution is maintained and recurrence relations are given explicitly, which may somehow give an efficient method.^46^ Hayat et al. advanced his research on the analytical solution of the fourth grade fluid, but between two fixed porous walls, considering the constant pressure gradient and the generating nonlinear problems which are further resolved for a series solution by the homotopy analysis method (HAM), where the results of the velocity and the shear stresses on the walls are obtained.^47^ Nadeem et al. analyzed the fourth-grade fluid with variable viscosity but with the consideration of flow and heat transfer characteristics, which demonstrates the two models Reynolds and Vogle, where the rigid cylinder is immersed at a constant pressure gradient with partial slip at the wall of the cylinder.^48^ Rasheed et al. explain the same fourth grade fluid on slip conditions taking the velocity into consideration, where they found the further complex nonlinear equation based on the hyperbolic sine function and the integral which is resolved with the new method Galerkin finite elements and the error analysis method.^49^

The evolution of digital computers in power and availability has produced an expanding utilization of practical numerical models in medication, natural sciences, business, medicine, and engineering to solve with more complexity and precision the models of the world. The formal scholastic region fluctuates from profoundly hypothetical numerical investigations involving the effects of Architecture on the implementation of definite algorithms. The Weighted Residual Method (WRM) is used in the engineering area for solving the differential equation of a boundary value problem analyzed by the Collocation, Subdomain, Least Square and Galerkin’s methods. Similarly, the Rayleigh–Ritz method is used to study the dimensional analysis in finding the approximation of the eigenvalue equation that is difficult to resolve analytically. In order to resolve this, we have to convert the differential equation into the weak form by using the trial function, especially in the method of Finite Element. Mirza et al. used the spatially adaptive grid-refinement method to solve the even-parity Boltzmann transport equation of second order by implementing the computer program ADAFENT (adaptive finite element for neutron transport). The program contains the K^+^ module, which covers the Lagrange polynomial for finite element formulation, and the Legendre polynomial, which is called an adaptive grid generator to determine the local gradient and residuals, and then finally a comparison is made between the spatial grid-refinement and the uniform meshing techniques.^32−35,50^ They further advanced their research from the variational principle of *K*_λ_^+^ to use the discontinuous function where the spatial variation of the angular flux has been based on a finite element while the Legendre polynomial has been used for directional dependence. A computer program has also been written, but only for one dimension. Different orders of angular supposition have been used to reduce the computing time. Then the result is compared with the exact solution as well as conventional continuous finite element solutions.^51^ Iqbal et al. used the cubic Lagrange polynomial in Galerkin’s finite element technique, which is a more accurate and efficient method to solve the second order boundary value problems as compared to the finite difference and spline method.^52^ He further proceeded with the idea of the Galerkin finite element, but on the weighted-residual formulation used to find the supposed solution of unilateral and contact-second order boundary-value problems. This method uses the piecewise linear approximation by using the linear Lagrange polynomial, which shows the best accuracy having low computational cost.^53^ Similarly, the concept of a BVP singular two point is also resolved by the Galerkin’s finite element, which changes the ODE consisting of a singular coefficient. This method is useful while reducing the partial differential equation into the ODE using physical symmetry, which shows the effectiveness, and the comparison is made of the numerical result with the exact result.^54^ Iqbal et al. further analyzed the one-dimensional flow of a pseudoplastic fluid with heat transfer by using the finite element techniques along with the exact solution of the fluid velocity and the fluid temperature. This shows that the Finite Element Method is more efficient as compared to the exact method with increasing values of the parameters.^55^ Iqbal et al. used the same Galerkin’s finite element technique on the weighted-residual to find the approximated solution but on the fourth order BVP. This method utilizes the piecewise cubic approximation, but using cubic Hermite interpolation polynomials i–e numerically having more accuracy and less computational cost as compared to the cubic-spline, nonpolynomial spline and cubic nonpolynomial spline methods.^56^ Iqbal et al. further use the Galerkin’s finite element of the fourth order BVP but also make a comparison between four methods which shows the efficiency of the Galerkin’s Finite Element Method. They compare the Galerkin’s finite element with the differential transform method, the Adomian decomposition method and the homotopy perturbation method with consideration of the Hermite interpolation polynomial.^57^ Iqbal et al. uses the spatially adaptive grid refinement technique based on the same Galerkin’s Finite Element Method to find the numerical solution of obstacle, unilateral and contact but of the second order BVP. This method uses the piecewise linear supposition utilizing the linear Lagrange polynomial.^58^ Iqbal et al. further advanced his research on Galerkin’s Finite Element Method, but on the third order BVP, and showed that the results are more accurate and efficient as compared to those of the quartic spline, quartic nonspline cubic spline finite difference, quintic spline, and quartic B-spline techniques. They further used the Galerkin’s finite element based on the weighted-residual on the second order obstacle problem. The technique has a piecewise quadratic shape function for checking the approximated solution for a spatially adaptive finite element grid, where a comparison of an adaptive refined grid with that of a uniform mesh is also made showing that the adaptive refined grid is superior to the other one.^59^

These investigations led to the development of colloidal electrolytes that improve the overall performance of batteries, including improved energy density, charge/discharge efficiency, and cycle life. In addition, rheology research contributes to the development of safer batteries by addressing issues such as dendrite formation and preventing short circuits and thermal instability. Studies of rheological properties also guide the formulation of optimized electrolyte compositions for specific cell designs to ensure compatibility with electrode materials and meet performance requirements. Understanding these properties is also important for maintaining a stable and effective electrode–electrolyte interface, which impacts the overall electrochemical performance of the battery. Additionally, rheological studies contribute to the development of colloidal electrolytes with properties suitable for efficient manufacturing processes, such as ease of handling, mixing, and application in battery manufacturing. Finally, insights into rheological properties will drive innovation in next-generation battery designs, leading to the development of batteries with improved properties such as flexibility, lightweight construction, and suitability for specific applications.^23,60−64^ Most of the numerical problems are related to a mathematical equation with a variety of quadratic equations, algebraic equations or in the form of differentiation or integral form which was optimized by the homotopy perturbation method^41^ or homotopy analysis method^44^ in order to solve as a proposed problem of the 1-dimensional, second order equation of thin film flow of a fourth-grade fluid down a vertical cylinder which is optimized by applying Galerkin’s Finite Element Method.

## 2 Methodlogy and Galerkin’s Finite Element Formulation

### 2.1 Reason for Selecting Galerkin’s Finite Element Formulation

The Finite Element Method (FEM) is a powerful numerical technique used in engineering and physics for solving Boundary Value Problems (BVPs). However, it may be ineffective under certain conditions, notably in situations where there is nonlinearity and systems of very high complexity. Its main capacity is to deal with and address materials’ nonlinearity. The fundamentals of physics, like Hooke’s law, the laws of thermodynamics, Fick’s law, etc., dictate how stress and strain are linearly related according to the Finite Element Method. Given that some materials undergo nonlinear deformations, such as yielding or strain hardening, it would be more fitting to use either the Finite Difference Method (FDM), the Finite Volume Method (FVM) or other sophisticated nonlinear finite element analysis techniques which do not make that assumption. Its other capacity is to deal with and address geometrical nonlinearity The Finite Element Method may be inadequate in instances of significant deformations and where nonlinear effects are the most prominent issue (e.g., buckling, contact problems). Sophisticated methods like the Total Lagrangian Formulation or the Updated Lagrangian Formulation ought to be employed, as these may drastically heighten the computational complexity.^50−54^

Galerkin’s finite element formulation is frequently selected because of its benefits and fit for the particulars of the problem for examining electrolyte behavior in lithium-ion battery systems. Although there are numerous other numerical methods existing for simulating fluid dynamics as partial differential equations (PDEs) based models, Galerkin’s approach has certain prizes when it comes to electrolyte modeling. Electrolytes used in lithium-ion batteries have a range of characteristics, including conductivity, density, and viscosity. By including changeable material characteristics into the finite element formulation, Galerkin’s technique offers a more accurate depiction of the behavior of the electrolyte. Here the techniques used for modeling of LIB electrolytes have been described in Table 2. Galerkin’s approach allows for both temporal and spatial adaptivity, facilitating a precise mesh. The established reputation of Galerkin’s approach in similar fields of inquiry gives researchers confidence in its accuracy and efficacy.^65−68^

**Table 2:** Techniques Used for Modelling of LIB Electrolytes

**Sr. no**	**Technique**	**Description**	**Ref**
1	Porous electrode theory	treats the porous electrode as a superposition of active material, electrolyte, and filler, with each phase having its own volume fraction. The material balances are averaged about a volume small with respect to the overall dimensions of the electrode but large with respect to the pore dimensions. This allows one to treat electrochemical reaction as a homogeneous term, without having to worry about the exact shape of the electrode– electrolyte interface	(20)
2	Concentrated solution theory	offers the affiliation between driving forces (such as gradients in chemical potential) and mass flux. The flux equation is then used in a standard material balance to account for the transient change of concentration due to mass flux and reaction	(21)
3	Ohm’s law	describes the potential drop across the electrode and also in the electrolyte. In the electrolyte, Ohm’s law is modified to include the diffusion potential	(22)
			(23)
4	Butler–Volmer equation	generally used to relate the rate of electrochemical reaction to the difference in potential between the electrode and solution, using a rate constant (exchange current density) that depends on the composition of the electrode and the electrolyte	(24)
			(25)
5	Galvanostatic polarization technique	developed to provide a simple yet rigorous method of obtaining transference numbers in nonideal polymeric electrolytes	(26)
			(27)
6	Molecular dynamics (MD) technique	a simulation technique in which assumed intermolecular potentials are used to calculate trajectories of a modestly sized collection of molecules. From such trajectories desired physical properties can be calculated. Many MD simulations have been performed for simple ion–water systems	(28)
			(29)
7	Poisson–Nernst–Planck (PNP) equations	continuum mechanical treatment of the electrolyte is done through these equations	(30)

### 2.2 Governing Equation and Galerkin’s Finite Element Formulation

**Figure 1:**
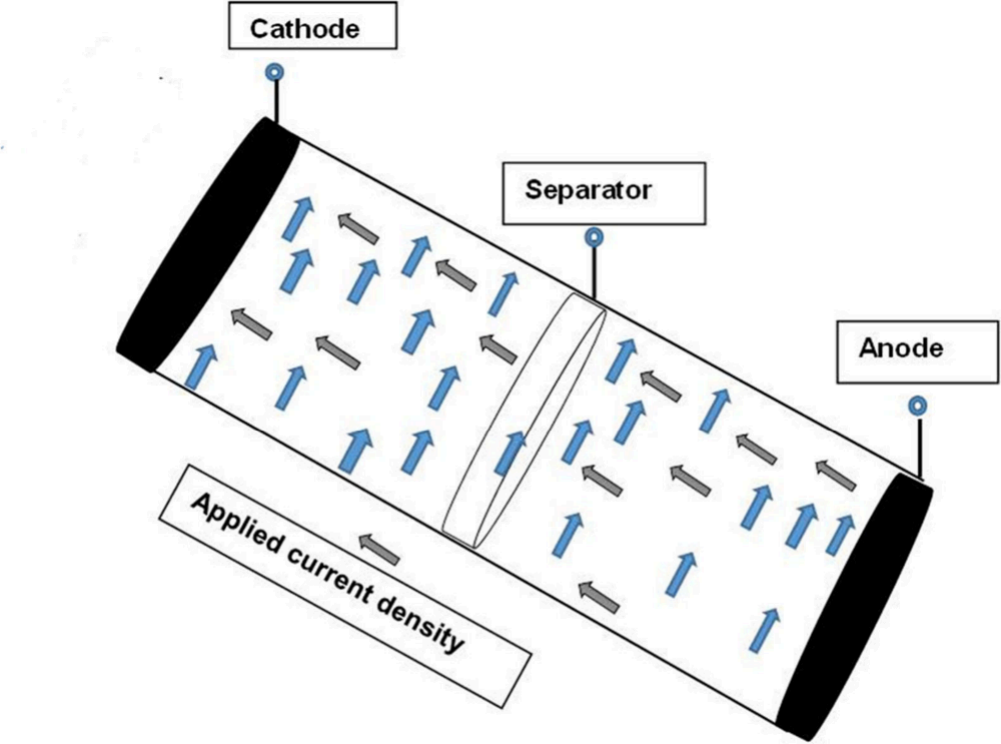
Flow geometry of electrolytes for lithium-ion batteries.

Consider an electrotype as a fourth grade fluid in Li-ion batteries dropping in, as given in Figure 1, an infinitely long vertical cylinder of radius *R*. The flow is considered to be in a thin, uniform axisymmetric film with thickness δ in contact with stationary air. In cylindrical coordinates we have^54^
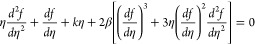
1The boundary condition is
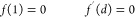
2
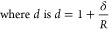
3

Dividing eq 1 by η we get
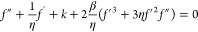
4

By rearranging eq 4, we get
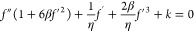
5
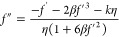
6Using quasi linearization for eq 4, we proceed as follows:
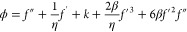
7Now differentiating eq 5 by *f′*, *f′′* we get
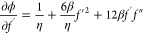
8
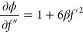
9
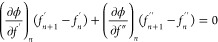
10Substituting the values of ,  and *f*_n_^′′^ in eq 10, we will find
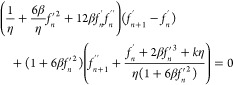
11
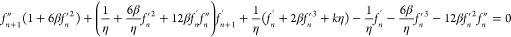
12By rearranging eq 12, we get
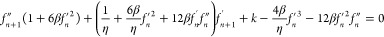
13Dividing the above eq 13 by (1 + 6β*f*_*n*_^′2^), we obtain
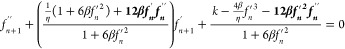
14
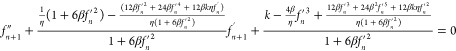
15By rearranging eq 15, we get
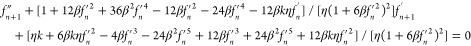
16
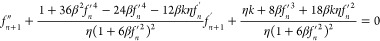
17

Now, let us apply the weighted residual method in Galerkin’s fashion. Multiplying eq 17 by *w* (a weight factor) gives
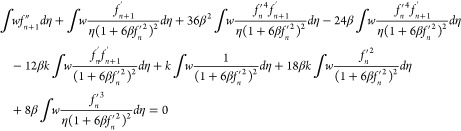
18
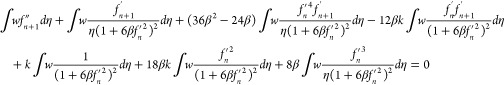
19

The above eq 19 is in strong form, and we have to convert it into weak form for Galerkin’s formulation:
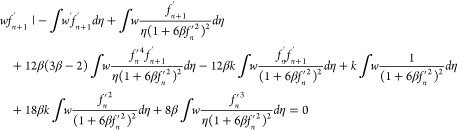
20
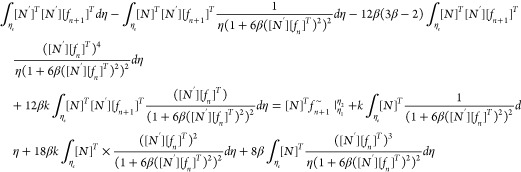
21
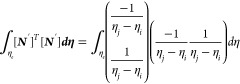
As *L* = *η*_*j*_ – *η*_*i*_, which is the Lagrange polynomial
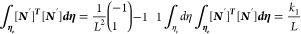
22where
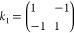
Similarly
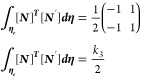
23where
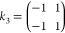
Now
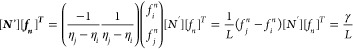
24where
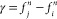
Similarly
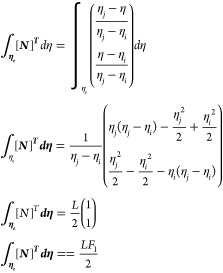
25where
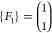
Now
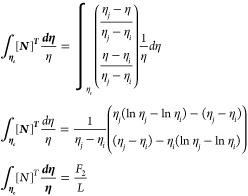
26where
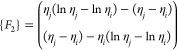
Now
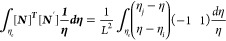

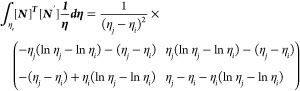

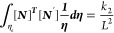
27where
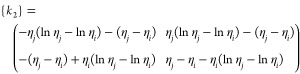


Substituting the values of eq 22 to eq 27 in eq 21 gives
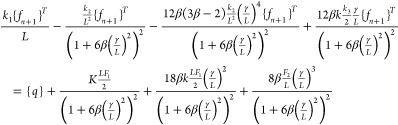
28
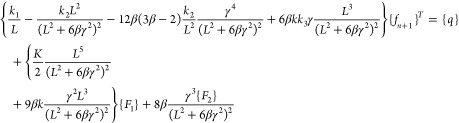
29
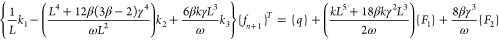
30
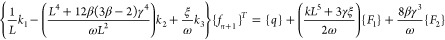
31
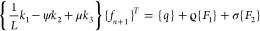
32
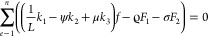
33
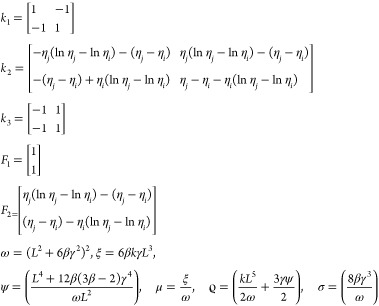
where *k*_1_, *k*_2_, and *k*_3_ are the stiffness matrices and *F*_1_ and *F*_2_ are force vectors and *q* is the vector with respect to the boundary conditions.

## 3 Results and Discussion

Numerical results were obtained by the derived formulation. MATLAB is used to implement the derived formulation of Galerkin’s Finite Element Method.

**Figure 2:**
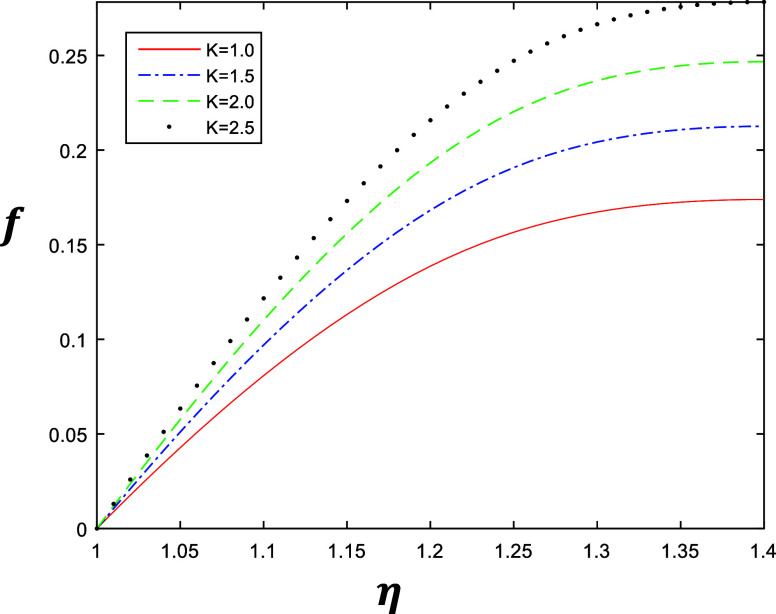
Velocity profile for different values of *k* with constant values of β = 0.5, δ = 0.4, and *R* = 1.

**Figure 3:**
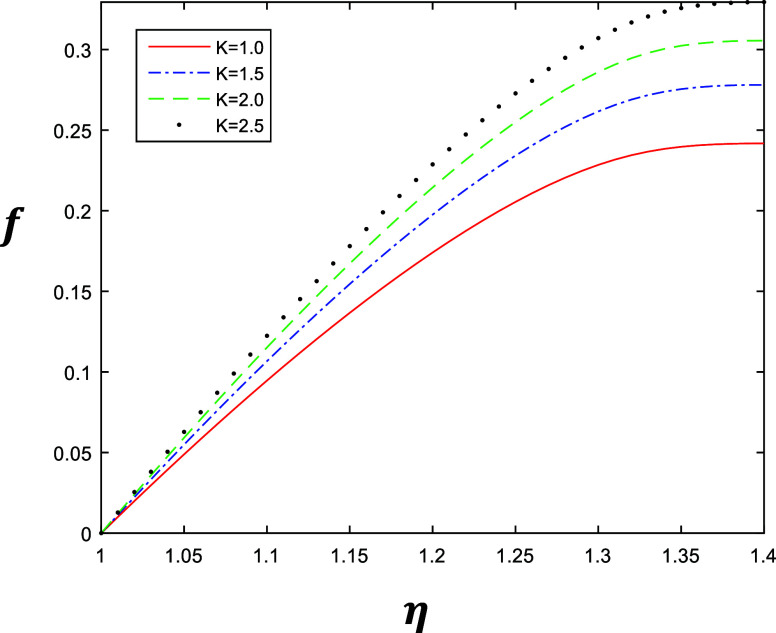
Velocity profile for different values of *k* with constant values of β = 1, δ = 0.4, and *R* = 1.

**Figure 4:**
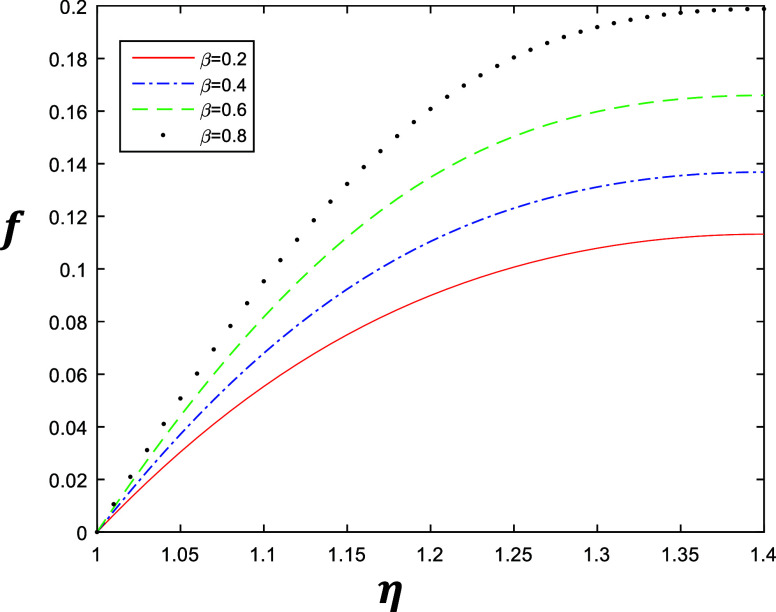
Velocity profile for different values of β with constant values of *k* = 1, δ = 0.4, and *R* = 1.

**Figure 5:**
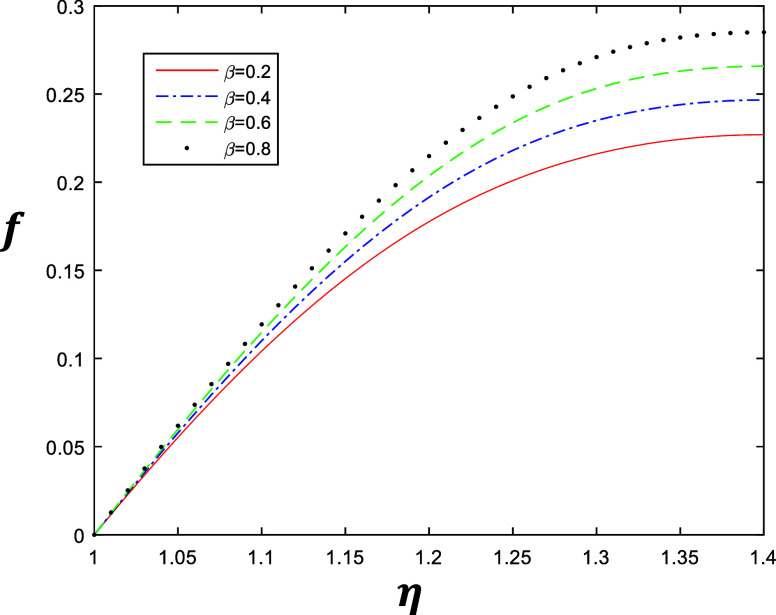
Velocity profile for different values of β with constant values of *k* = 2, δ = 0.4, and *R* = 1.

**Figure 6:**
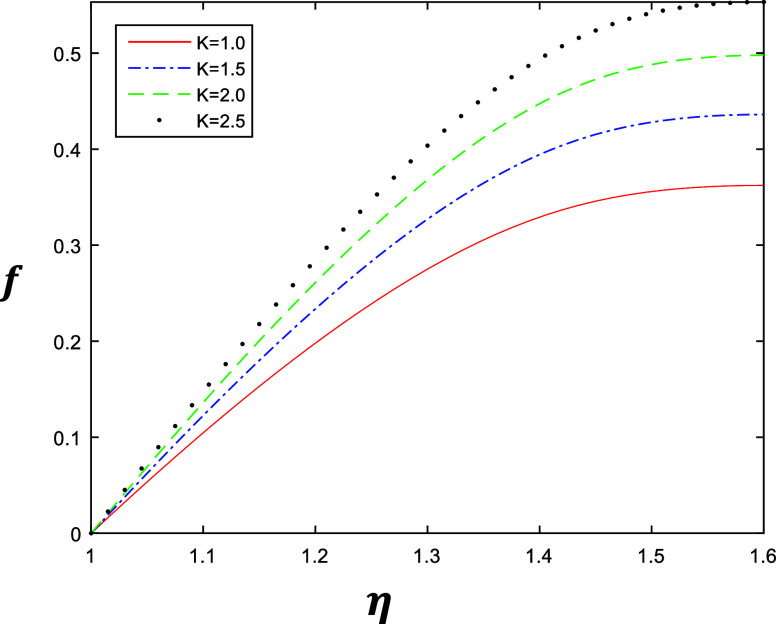
Velocity profile for different values of *k* with constant values of β = 0.5, δ = 0.6, and *R* = 1.

**Figure 7:**
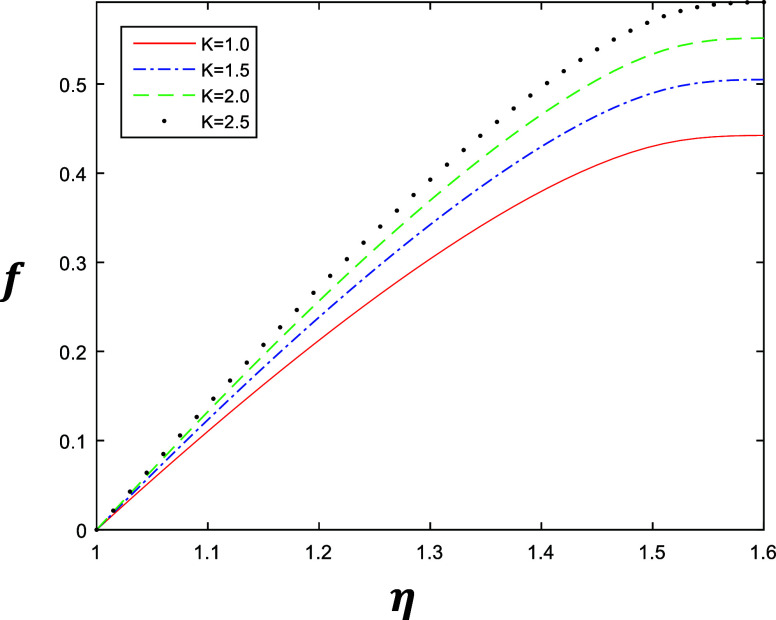
Velocity profile for different values of *k* with constant values of β = 1, δ = 0.6, and *R* = 1.

**Figure 8:**
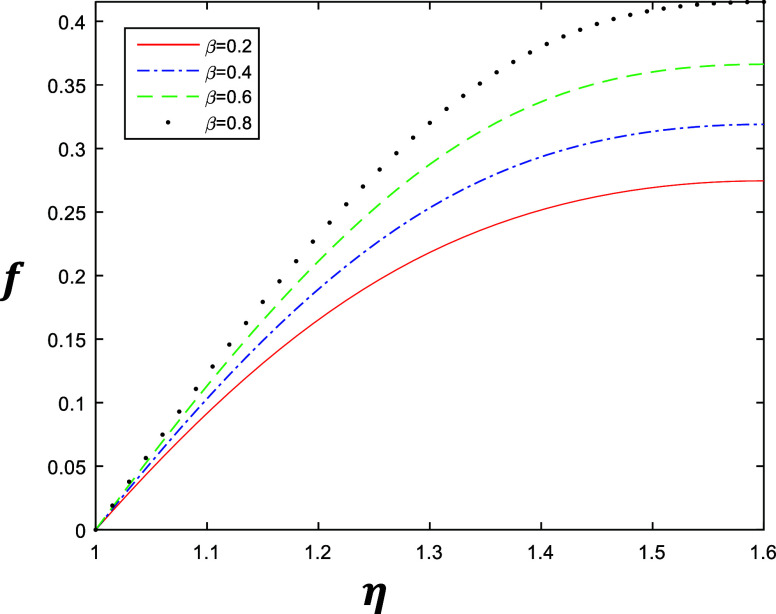
Velocity profile for different values of β with constant values of β = 0.5, δ = 0.6, and *R* = 1.

**Figure 9:**
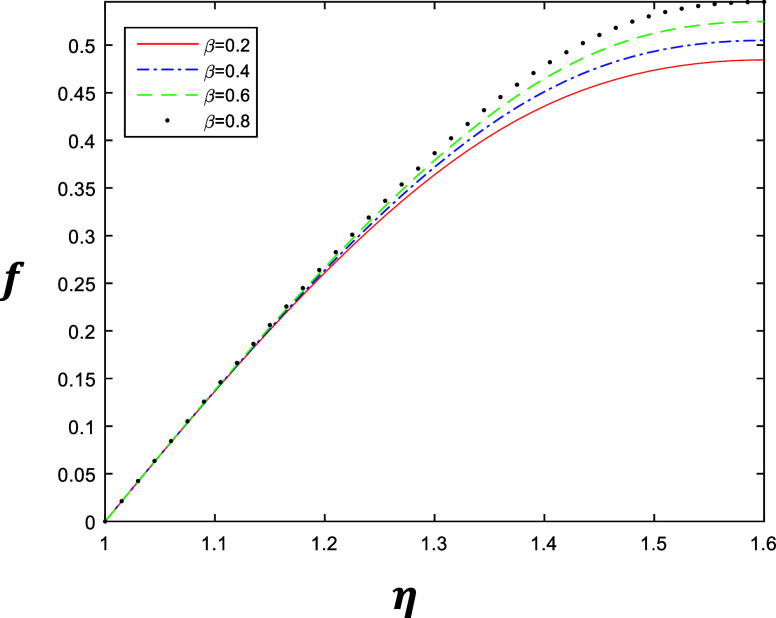
Velocity profile for different values of β with constant values of β = 1, δ = 0.6, and *R* = 1.

It is noticed that as the number of iterations increases, the approximate solutions are going to be more precise and accurate in considering the influencing parameter β, δ, *R*, and *k*. The graphical results obtained are shown in Figures 2–9. In Table 3, the properties of the Li-ion batteries used in this section are given.

**Table 3:** Properties of Li-Ion Batteries Used in Modelling and Simulation

Properties		Refs
No. of cells in the battery pack	24	(3−7)
Configuration of the battery pack	6 × 4	
Flow arrangement of the cooling duct	Z-type	
No. of fluid flow passages	38	
Cell shape	Cylindrical	
Size of each cell	18 mm dia, 65 mm length	
Cell voltage	3.7 V	
Cell capacity	2600 mAh	
Charge current	1 A	
Cell backup time	3.9 h	
External coating	Nickel	
Operating temperature range	298 K to 333 K	

The numerical results are shown in Figures 2–5, which describe the velocity profile of the fourth grade fluid for energy storage obtained by solving eq 1 after applying Galerkin’s Finite Element Method with emerging parameters *k*, β, δ, and *R*. Figure 2 and Figure 3 represent the variation of *k* ∈ {1, 1.5, 2, 2.5} at β = 0.5 and β = 1 with a constant value of δ = 0.4 and *R* = 1, which describe the behavior of fluid flow relative to *k*. The flow of the fluid is gradually increasing when we increase *k*. Similarly Figure 4 and Figure 5 represent the variation of β ∈ {0.2, 0.4, 0.6, 0.8} at *k* = 1 and *k* = 2 with constant values of δ = 0.4 and *R* = 1, which describe the behavior of fluid flow relative to β. The flow of the fluid is gradually increasing when we increase β.

It may be examined from Figures 6–9, which describe the same behavior of the velocity profile of the fourth grade fluid obtained by solving eq 1 after applying Galerkin’s Finite Element Method with the emerging parameters *k*, β, δ and *R*, that the value of δ is 0.6 as compared to 0.4 with *R* = 1. Figure 6 and Figure 7 represent the variation of *k* ∈ {1, 1.5, 2, 2.5} at β = 0.5 and β = 1. Similarly, Figure 8 and Figure 9 represent the variation of β ∈ {0.2, 0.4, 0.6, 0.8} at *k* = 1 and *k* = 2, as depicted in the above section. The results indicate the accuracy of the solution after the formulation of Galerkin’s Finite Element Method.

The technique of Galerkin’s Finite Element Method is applied in eq 1 to find the velocity profile of a fourth-grade fluid for energy storage down a vertical cylinder. Similar problems were solved using with the comparison of homotopy analysis method (HAM) and the homotopy perturbation method (HPM). It may be examined from Figures 2–9 that as the number of finite elements increases over the length *L*, the graph is going to be more straight, which shows the efficiency of Galerkin’s finite element. Our result is also compared with the existing parameters *k*, δ, *R* and β. Therefore, we conclude that fluid flow is increasing with the increasing values of *k* and β while holding the other parameters fixed. Our results indicate superior performance of our approach in accurately approximating the solution.

Velocity, fluid concentrations, and viscosity are interlinked to transport of electrolyte and the thermal phenomenon inside the battery system. As temperature within the battery system increases, the viscosity of the electrolyte drops down and, hence, the velocity increases. More rapid and greater increases in velocity and fluid concentrations are due to less thermal efficiency of electrolyte, and slower increase in velocity is due to more thermal efficiency of the electrolyte.

## 4 Exploring the Practical Purposes of Fluid Parameters in Lithium-Ion Based Battery-Systems via Computer Code

The investigations that are emblems of chemistry and fluid mechanics are always fascinating because of new discoveries. Some parameters of fluids, such as fluid pressure, viscosity, and velocity, all have a significant impact on the behavior and performance of electrolytes in lithium-ion batteries. It is arguable how changing these settings may affect battery performance and efficiency. During charge and discharge cycles, improved mass transport within the electrolyte facilitates the passage of ions between the electrodes. This enhances battery performance, particularly with regard to the effectiveness of charging and discharging. However, decreasing fluid velocity can impede ion movement and diminish mass transfer, which can have an impact on the battery’s overall efficiency. Controlled lower rates, however, could be preferable for some battery types or uses. Viscosity: Greater viscosity can impact the velocity of electrolyte flow by limiting it and raising internal resistance. A higher viscosity can have an impact on the pace of ion diffusion by limiting the flow of the electrolyte and raising internal resistance. Battery performance may suffer as a result, particularly in terms of power density. On the other hand, reduced viscosity promotes more even ion mobility in the electrolyte, which can accelerate the charging and discharging of batteries. On the other hand, issues like diminished structural stability and electrolyte leakage may arise if the viscosity is too low. Higher liquid pressure has a beneficial impact on the ion transport and diffusion rates within the electrolyte, which raises the rates at which ions charge and discharge.^57−63^

Because of this, the battery is more appropriate for sequences that require rapid energy proclamation. On the other hand, low fluid pressure can decrease mass transfer and impede ion mobility, which can have an impact on the battery as a whole. Increased fluid pressure has a beneficial effect on the electrolyte’s ion transport and diffusion rates. This may result in faster rates of charging and discharging, which would make the battery more appropriate for uses requiring rapid energy release. Ion mobility and mass transfer may be slowed down by decreasing the fluid pressure. Specific necessities and essential possessions of ionic electrolytes for LIB propositions have been widely deliberated. Furthermore, the transference machinery of Li-ions has been methodically enlightened to design a safer battery system for long-term functionalities.^64,63−65,69^

**Figure 10:**
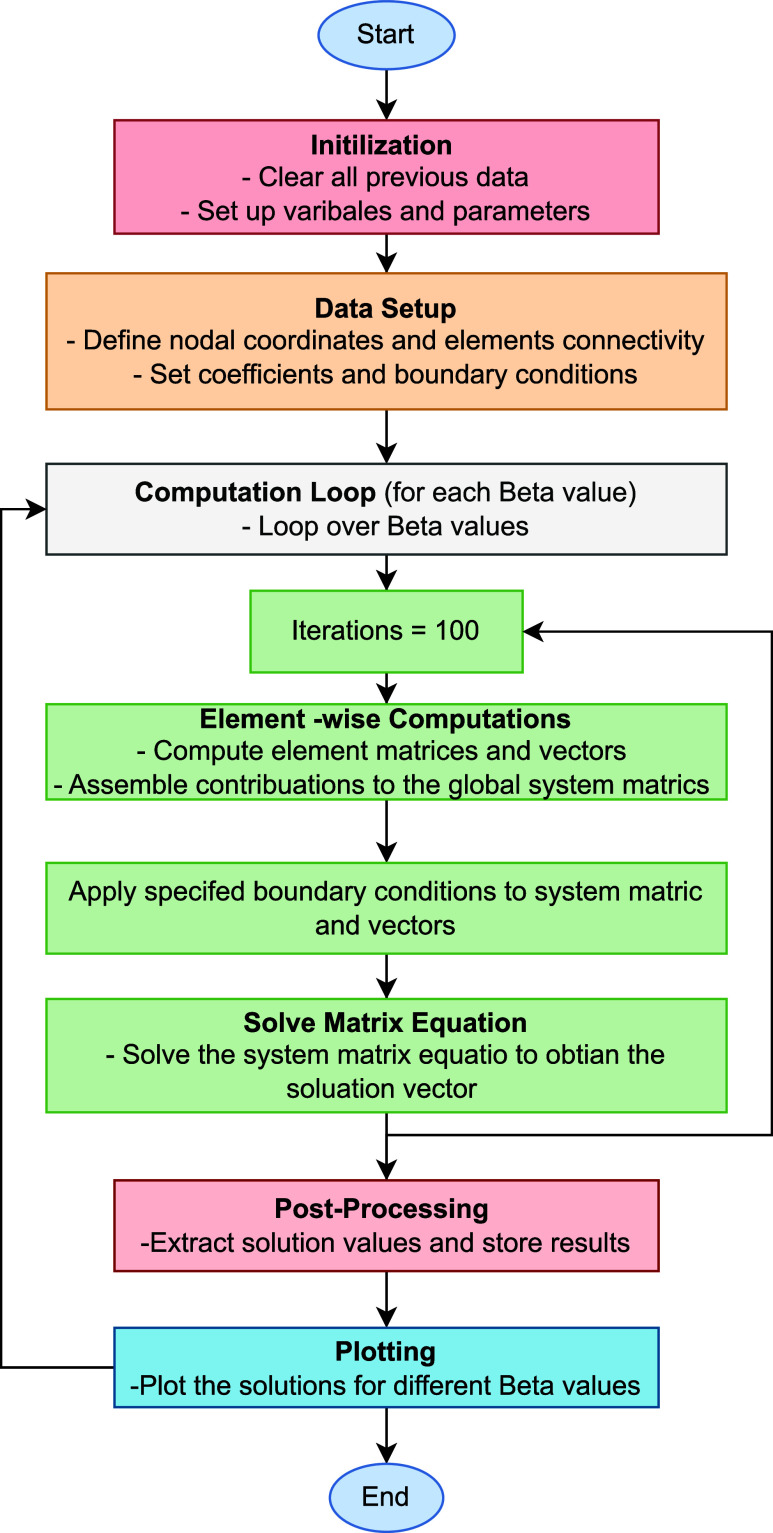
Flowchart of MATLAB code.

We have given MATLAB code in Figure 10 that explains how we have used weighted functions and other formulations. This is a robust code of a Galerkin solution that was used in different ways by others as well. The *space-time-based parametrization* of these steady-state and transient problems is deliberated with the steadied assorted Galerkin method. Furthermore, this method may be pragmatized in any category of boundaries (curved and irregular) without being compromised for correctness and precision in semisolid fluids.^56,70−73^ The future work contains a parallel solution of Darcy-based ionic flows in a discrete atmosphere for distributed systems by the use of advanced-array CPUs. Moreover, the authors strategize to implement the parallel Darcy flow algorithm for numerous filaments by exploiting well-organized CPU memory supervision.

## 5 Summary and Conclusions

- The Finite Element Method (FEM) has a very deep impact in the fields of energy-engineering, computational designing and mathematical physics and is utilized to resolve the complexity of these differential equations for rheological properties of colloidal electrolytes such as thin film flow of a fourth grade fluid for lithium ion (Li-ion) batteries. The FEM is generally referred to for structural analysis, heat transfer, fluid dynamics, electromagnetic potential and automobile engineering problems, which requires that the Solution of boundary value problems (BVP) exist in partial differential equations. The outcome of the above work is also compared with the current electrolytes’ parameters like *k*, δ, *R* and β. Therefore, we conclude that electrolytes flow is growing with the increasing values of *k* and β while holding the other parameters fixed. Our results specify a greater performance of our method in precisely approximating the solution.
- In this work, the rheological features of electrolytes can be better explicated by the training of the finite element process, which enables spatially resolved simulations and extensive analysis.
- In this present work, Galerkin’s finite element formulation has been displayed to resolve the fourth grade fluid BVP. Numerical results can be found by implementing the formulation using MATLAB on different parameters. Results were seen to be fantastic with this approach. The outcomes acquired after implementing the FEM are superior to those from other exiting techniques. Moreover, we found that fluid flow is increasing with the variation of β as well as with the variation of *k* at different parameter values of δ and *R* from Figures 2–9. The numerical outcomes acquired strengthen the assumption made by numerous investigations that the productivity of Galerkin’s Finite Element Method gives it a lot more extensive applicability.
- Through the present work, the practical propositions that are being expounded by exploring the rheological characteristics of colloidal electrolytes for lithium-ion batteries are several, including refining energy density, charge–discharge efficacy, cycle life and the complete demonstration of batteries. Rheological resources also control the preparation of enhanced electrolyte configurations custom-made to specific battery projects, ensuring compatibility with electrode resources. Furthermore, rheological studies encourage the advance of safer batteries by addressing issues such as precipitate development, averting short-circuits and thermal uncertainties.
